# Role of servitization in transitioning from scarcity to abundance paradigm

**DOI:** 10.3389/frma.2022.1016432

**Published:** 2022-09-29

**Authors:** Manpreet Hora

**Affiliations:** Scheller College of Business, Georgia Institute of Technology, Atlanta, GA, United States

**Keywords:** servitization, scarcity, abundance, digital products, business models

## Abstract

Servitization refers to firms that sell an “outcome-as-a-service”, rather than just a physical product. In this study, we first examine how servitization has enabled companies such as Netflix to disrupt industries and transition from offering finite products to delivering relatively abundant services? Second, as firms embark upon servitization, value propositions become much less related to scarcity. This leads to the second research question: what are the value propositions for consumers when the paradigm shifts from ownership to usership? For both these questions, we highlight examples such as Netflix, Amazon Web Services (AWS), and Philips to emphasize on value propositions for the consumer as enhanced customer experience through customization, convenience, and co-creation. Further, we expand on the considerations warranted that include the role of technology, data, and analytics, distribution models for physical versus digital products, and challenges in creating servitization in business models.

## Introduction

Servitization refers to firms that sell an “outcome-as-a-service”, rather than just a physical product (Karmarkar, 2021; Mutha et al., 2022). Software-as-a-service, which allows software products to be distributed *via* the internet, is one of the most common and influential examples of this phenomenon. The digitization of things utilizing cloud computing platforms and cyber-physical systems has, for several products, transformed “the state of scarcity into that of abundance” (Tronvoll et al., 2020, p. 301).

In this study, we focus on two objectives and first examine (i) *how servitization has enabled companies such as Netflix to disrupt industries and transition from offering finite products to delivering relatively abundant services*.

To address this initial objective, we first highlight Netflix as a mini-case study and the modes of expansion the company has embarked upon. Netflix started as a subscription service that would allow users to have DVDs delivered to them *via* mail. Upon return, a user could select new titles to replace the ones recently returned. That model has since transformed with the advent of digital streaming services and has created a supply where content is not scarce but in contrast, exists in abundance. When a customer is interested in a particular piece of digital content, there is no longer a risk of that item being “out of stock,” or already rented to a different customer. Rather, the digital product exists in near-infinite abundance *via* the Internet.

(ii) As firms embark upon servitization, value propositions become much less related to scarcity. This leads to a second research question: *what are the value propositions for consumers when the paradigm shifts from ownership to usership?*

To address this objective, we continue with the example of Netflix and introduce other examples such as Amazon Web Services (AWS) and Philips that highlight value propositions for the consumer as enhanced customer experience through customization, convenience, and co-creation. These considerations will include the role of technology, data, and analytics, distribution models for physical versus digital products, and challenges in creating servitization in business models (Sawhney et al., 2003; Kastalli et al., 2013; Visnjic et al., 2016; Coreynen et al., 2017; Sjödin et al., 2020).

## Servitization and products and services that exemplify transitioning from scarcity to abundance

The definition of servitization has moved from enhanced service offerings with the sale of a product to delivering the outcome of a product as a service (Karmarkar, 2021). For example, firms such as Netflix and Spotify have steered the entertainment industry and its customers from purchasing CDs and DVDs to streaming video and audio content.

### Servitization of digital products in business to consumer markets: Netflix

When Reed Hastings and Marc Randolph founded Netflix in 1997, Blockbuster was the largest company in the video rental business, with six billion $ in annual revenue. Despite Blockbuster's dominance, customers were dissatisfied with the company's cumbersome late fee structure and a lack of available titles, especially for new releases. At one point, late fees made up 70% of Blockbuster's profit. At the same time, due to scarcity (unavailable inventory or stockouts), only 20% of customers could rent the movie they had in mind when entering the store. At its founding, Netflix was well-positioned to create abundance in the form of content availability in a space plagued by scarcity and customer dissatisfaction. Amazon's success in the book market had inspired online retail in the movie rental industry, especially since the newly invented DVD was relatively inexpensive to ship. By 2000, Netflix had grown substantially, but interestingly, Blockbuster showed no signs of feeling threatened and was insistent that consumers would “never give up their video stores.” The company even turned down an early opportunity to purchase Netflix outright for $50 million.

Of course, the subsequent downfall of Blockbuster (partly at the hands of streaming services, mainly Netflix) is well-known. However, the story of Blockbuster's competitive response, and its near-victory over Netflix, is lesser-known. In 2004, Blockbuster Online launched with an operating model nearly identical to that of Netflix. It had more titles, no late fees, was less expensive than a Netflix subscription, and was backed by the iconic Blockbuster name. Within nine months, the service garnered one million subscribers. The subsequent launch of Blockbuster Total Access added several additional features that consumers valued, including the ability to return or exchange movies in Blockbuster stores instead of mail for no extra charge. After this launch, Netflix lost subscribers for the first time, and most new subscribers in the video rental market were signing up with Blockbuster instead. Blockbuster had succeeded in creating even more product abundance by merging its retail and online operations. Customers could rent and return movies *via* mail or in-store. It was on track to catch up to and surpass Netflix's performance in terms of market share.

This initial success was promising, but Blockbuster soon realized that creating both a mail-order distribution system and an online presence is costly. While developing these new services, Blockbuster would lose money until it reached a certain subscriber threshold. Unfortunately, the company also had one billion $ in debt at the time. It could not afford to continue investing in Blockbuster Total Access while simultaneously paying down its debt. Given this debt situation, activist investor Carl Ichan pushed the company's board and management to spend less on and ultimately pull out of Blockbuster Total Access. With a significant debt repayment due in 2009, Blockbuster filed for bankruptcy.

Netflix continued to innovate to create further abundance for its customers first by moving into online streaming and then by creating original content (Jenner, 2018). The amount of content that Netflix subscribers could access at the click of a button was growing significantly. Its proprietary algorithms allowed it to track user interests and recommend new shows or movies the subscriber might enjoy. This detailed data unlocked enormous value for Netflix and created a positive feedback loop related to value creation. As Netflix learns more about the type of content users are interested in, it can develop more content that is increasingly likely to satisfy customers. The shift toward an abundance of data in the streaming space has allowed Netflix to create more value for itself and its subscribers.

Table 1 summarizes how Netflix in the digital business-to-consumer (B2C) products space helped transition the business model from scarcity to abundance. The table also illustrates the updated value propositions for the customer and the operational capabilities of Netflix to deliver such value. In sum, Netflix began as a mail-order DVD service, allowing access to an experience that was slightly more convenient than the experience received at brick-and-mortar stores such as Blockbuster. However, its inventory and distribution model still limited its ability to move away from scarcity. It was not until Netflix moved to an online streaming model that it could capitalize on abundance and offer new value propositions to customers. Netflix's content-filtering algorithm uses data and digitization to recommend content to customers, and its cross-device continuity creates a seamless user experience. Netflix would not have achieved such meteoric success without this shift to digitization.

**Table 1:** Digital business to consumer (B2C) products.

**Business model transformation**				
**Company name**	**Scarcity**	**Abundance**	**Value proposition**	**Organizing to deliver value**
Netflix	• In-store media rentals	• Subscription-based video streaming	• Recommendation engine	Development of *SaaS business models*.
	• Mail order DVD rentals			
				All firms are invested in sophisticated *technological infrastructure* (example: recommendation engine development) and *IT services*.
			• Cross-device continuity	
Spotify	• CD and cassette sales	• Subscription-based audio streaming	• Media accessibility	
	• Digital MP3 downloads		• Recommendation engine	
Wikipedia	• Physical encyclopedia sets	• Crowdsourced online encyclopedia	• Ease of access and efficiency	
				*Customer service* is of the utmost importance.
Microsoft 365	• Individual software copies	• Subscription-based, downloadable software products	• Cross-device continuity *via* the cloud	

### Other digital business to consumer (B2C) products

Similar versions of the Netflix story have played out in other companies, including Spotify, Wikipedia, and Microsoft. As Table 1 shows, technology has enabled these firms to transform typical physical consumer goods into digitized products—from CDs to encyclopedias to physical software licenses. These companies have all had to reorganize their operations to capitalize on opportunities driven by abundance and deliver value. Companies going through this transformation moved toward software-as-a-service (SaaS) business models and invested in significant technological upgrades. At this time, they also began to increase their focus on customer service and data security (Desai, 2013). With the shift to digitization, these aspects became increasingly crucial because customer value is no longer derived from scarcity.

The development of digital and crowdsourced encyclopedias is a particularly notable example. Since the early eighteenth century, physical encyclopedias have been in circulation, often employing sizeable editorial staff and receiving updates every few years. However, by the late twentieth century, encyclopedias began to be published digitally. A prime example was Microsoft's Encarta product, a digital encyclopedia distributed *via* CD-ROM that launched in 1993. This transition to digitization benefited users in several ways. Digital encyclopedias are portable, more easily searchable, and can link to supplementary digital media such as videos. Perhaps most importantly, they are dynamic, meaning the user does not need to wait several years for updated information. In theory, publishers would also benefit from digitization because it reduces costs. However, it also required a drastic operating model shift (Greenstein, 2017). Due to these challenges, after 1995 while Microsoft's Encarta “thrived,” Encyclopædia Britannica troubles “multiplied” (Greenstein, 2017). Finally, with the popularity of the internet and the abundance of information, crowdsourced encyclopedias such as Wikipedia emerged as Encarta closed shop in 2009 (Greenstein and Zhu, 2018). The move toward an open-source digital encyclopedia such as Wikipedia delivers even more value for users, despite the drastic impact that its non-profit business model had on the encyclopedia business overall.

### Digital business to business (B2B) products

A similar transformation occurs for business-to-business (B2B) products. These businesses shift from a model of scarcity to abundance by digitizing one or more of their existing products or developing new products that depend on digitization. Amazon Web Services (AWS), NCR, PayPal, and Littler have all leaned into digitization to create an environment of abundance where value is not driven by scarcity. Table 2 depicts these examples. Interestingly, Littler is an example of a business that primarily offers legal services, a profession that is typically highly dependent on human capital. The company successfully built software to automate many high-frequency and low-sophistication tasks that individual staff members typically performed. This development helped shift the company from a scarcity model to a model of relative abundance. Because many aspects of service delivery had been digitized, the company's offerings were no longer as limited by staff availability, and Littler could effectively serve even more clients.

**Table 2:** Digital business to business (B2B) products.

**Business model transformation**				
**Company**	**Scarcity**	**Abundance**	**Value proposition**	**Organizing to deliver**
**name**	**Ownership**	**Usership**		**value**
AWS	• On-premises servers and IT resources	• Cloud-based infrastructure	• Reduced on-site energy usage and maintenance	Development of *B2B SaaS business models*.
NCR	• Physical cash registers	• Banking and sales (POS) technology	• Connection with customers	All firms are invested in sophisticated *technological infrastructure* (example: cloud development) and *IT services*.
PayPal	• Physical money transfers	• Internet-based money transfers	• Security	
			• Ease of use	
Littler	• Legal services dependent on attorneys and specialized employees	• Automation and analytics “unbundle” service offerings	• Automated and data-driven business insights • Decreased costs	*Customer service* and *data security* is of the utmost importance.

Although both B2C and B2B have shifted to digitization, thus enabling abundance, it is essential to examine the two business models differently (Mutha et al., 2022).

Take the story of Amazon Web Services (AWS) as an example of B2B. Interestingly, Amazon initially developed the technology that became AWS internally. In the early 2000s, Amazon was experiencing rapid growth and was facing problems with scaling. The company intended to create a new development product that other retailers could utilize to list their products online using Amazon's infrastructure. However, before the company could develop this product, it needed to streamline its internal systems. The result was a new infrastructure service for internal use. During a 2003 retreat, the Amazon executive team realized that the service they had built for internal use could also bring value to external users. This realization was the beginning of AWS. AWS offered several value propositions to users by developing new internal core competencies. Most importantly, businesses no longer needed to be hindered by the limitations of on-premises servers. Cloud computing offers abundant storage and operational capacity for businesses of all sizes (Kushida et al., 2015).

AWS allows companies to move otherwise on-premises processes and activities, such as data record storage, into the cloud. This offering makes those necessary activities less costly and more reliable for the consumer, which in this case is a corporation. Here, users pay to use AWS's infrastructure and data centers to do their computing and only pay for the computing that they use. Instead of installing large local storage units and local processing in their facility, businesses can use AWS's cloud computing to store information or process requests. With AWS's scale, they can typically offer it cheaper than a business could install and operate on its own. Additionally, AWS's cloud computing can be scaled up and scaled back as needed. In short, as companies begin or start to grow, it makes sense to engage AWS's cloud computing instead of investing in large amounts of storage or hardware. While this strategy reduces the upfront cost for computing infrastructure for such client companies, engaging AWS may potentially increase recurring costs down the line. But that potential increase in cost is often significantly lower contingent on the business needs and the scale of the company. So, while both B2B and B2C business models create value for consumers, they do so in different ways *via* direct and indirect consumer benefits.

## Commonalities in the servitization of digital product business models

Four basic commonalities underlie servitization. First, the shift from scarcity to abundance also brings about a shift from ownership to usership of the underlying product, and thus, the pricing is more based on a fee structure. For example, the customer pays a fee (based on usage, subscription, etc.). Second, this also changes the characteristics of contracts such that the arrangements can become performance-based and/or based on the degree of customer involvement (Kastalli and Van Looy, 2013; Guajardo, 2018). Third, the focus transitions from the underlying product to customer value. That is, a customer's valuation of a product lies in the benefits and utility that the customer derives not only from the product itself but also from the underlying process to access the product. Typically, scarcity enhances the value of a product. In contrast, in an environment of abundance, the underlying process enhances the value of the product. Fourth, an inherent reorganization is required of the firm's operations and in some cases, a drastic operating model shift.

For example, Netflix provides customized content recommendations to users that contribute to customer value (Gomez-Uribe and Hunt, 2015). Netflix has two primary algorithms—a “collaborative filtering” algorithm that recommends existing content to users and a “content-based” algorithm that notes preferred content characteristics. Havens (2018) explores the impact of these algorithms on production decisions. When Netflix first entered the original content space, it utilized its content-based algorithm to determine the type of content that would be most successful on the platform. The algorithm indicated that users would be most interested in a political drama starring actor Kevin Spacey and directed by David Fincher. Thus, *House of Cards* was born and became one of Netflix's greatest hits.

Leveraging an algorithm to influence production decisions represented a significant shift in the entertainment industry. This shift was a direct result of digitization and the abundance of consumer data. As Havens (2018) points out that “one major change that has taken place for media industry workers at all levels is a shift from an era of scarcity of audience data to an era of overabundance” (p. 8). Netflix's digitization resulted in an abundance of data on user preferences that the company can leverage to make production decisions and content recommendation decisions. In moving from DVD mail orders to streaming content, Netflix had to reorganize its operational structure to collect this data and use it to create value for customers. Ultimately, customers received many additional benefits that resulted from this abundance of data. When Netflix determined how to offer content that users truly desired, the perceived value of a Netflix subscription increased dramatically.

## Opportunities with servitization

Philips^1^, a major player in the healthcare, consumer lifestyle, and lighting industries offers not only lightbulbs but has expanded and transitioned into offering lighting-as-a-service (LaaS). Typically offered to businesses such as large office buildings, warehouses, hotels, hospitals, and airports, this service offering outsources all setup and maintenance required with the lighting system. The transition in the industry is viewed as moving “from illumination-based applications to data-enabled services that offer a rich end-user experience. Data transmission through visible light spectrum will even complement existing data transfer technologies like Wi-Fi, and augment indoor connectivity”^2^. Accordingly, Philips installs additional sensors and uses data and analytics to reduce power usage when lighting is not needed. The intent of the offering is to reduce the Total Cost of Ownership (TCO) of the lighting products for the client. TCO considers costs associated with a given offering. Philips offers light as a service through a monthly subscription cost. They then manage the lighting fixtures and apply additional data analytics to reduce energy consumption throughout the building. The service is priced so that the TCO of the service is lower than the previous TCO of simply replacing lightbulbs when they burn out. The customer receives lower costs due to reduced energy consumption, maintenance, and purchasing logistics. Philips can accrue higher, more predictable revenues and has created a “stickier” customer with a higher switching cost when compared to their competitors (Porter and Heppelmann, 2015).

The additional opportunity of offering LaaS for Philips is something critical for our existing resource-scarce times: the circular economy. The circular economy is based on three principles: eliminate waste and pollution, circulate products and materials, and regenerate nature^3^. It builds on the notion of Industrial Ecology, a multidisciplinary field that highlights the importance of systems thinking when designing products from “cradle to grave.” As with servitization, the manufacturers retain ownership (Lay et al., 2009), thus, with Philips retaining control of the product it can reclaim valuable material at the end of life of the product. Philips Lighting's head of sustainable innovation Anton Brummelhuis points out that focusing on the circular economy, through servitization, “maximizes the reusability of products” and “instead of heading to a landfill, we have to make sure that products and raw materials come back to the economy. And we do this by maintaining the value. We have to minimize the destruction of value.”^4^

Thus, while servitization creates an abundance of real-time information through its Internet of Things (IoT) platform for Philips' clients so that they can drive efficiencies and provide more effective decisions^5^, it also creates an opportunity to reduce the utilization and the consumption of scarce resources (Spring and Araujo, 2017; Örsdemir et al., 2019).

## Challenges in creating servitization in business models

The challenges in creating servitization in a business model cannot be overlooked. First, data integrity becomes crucial. For example, Netflix in its 10-k in 2019, mentions among its significant potential risks, “any significant disruption in or unauthorized access to our computer systems or those of third parties that we utilize in our operations, including those relating to cyber security or arising from cyber-attacks, could result in loss or degradation of service, unauthorized disclosure of data, including member and corporate information, or theft of intellectual property, including digital content assets, which would adversely impact our business.”

Second, creating a successful servitization model entails an interdependent supply network. For example, as a B2C digital product provider, Netflix depends on a B2B provider, Amazon Web Services (AWS) for its cloud services. Netflix in its 10-k in 2019, mentions that “we rely upon Amazon Web Services to operate certain aspects of our service and any disruption of or interference with our use of the Amazon Web Services operation would impact our observations and our business would be adversely impacted^6^.”

Similarly, Amazon also mentions in its risks emanating from operating AWS, “we could be harmed by data loss or other security breaches: Because we collect, process, store, and transmit large amounts of data, including confidential, sensitive, proprietary, and business and personal information, failure to prevent or mitigate data loss, theft, misuse, or other security breaches or vulnerabilities affecting our or our vendors' or customers' technology, products, and systems, could: expose us or our customers to a risk of loss, disclosure, or misuse of such information; adversely affect our operating results; result in litigation, liability, or regulatory action (including under laws related to privacy, data use, data protection, data security, network security, and consumer protection); deter customers or sellers from using our stores, products, and services; and otherwise harm our business and reputation^7^.”

Third, while the servitization of digital products has created business models of attaining abundance where there was earlier existence of scarcity, it does not imply that the resources to create abundance for the customer are also abundant on their own. For example, resources such as infrastructural platforms, computing power, data storage, and the required human talent can be scarce, and firms can accrue scarcity dividends by utilizing these resources efficiently (Blevins, 2011; Mullainathan and Shafir, 2013). Moreover, while firms such as Netflix compete on their digital services and platforms, content also plays a critical role. This was recognized by both Netflix and large production companies such as Disney that were providing its movies and Pixar's titles to Netflix. In 2017, Disney announced that it has decided to pull its content from Netflix by end of 2018 and launch its own streaming service in 2019.^8^

The launch of the streaming service, Disney+containing Disney original movies, Pixar titles, Marvel movies and TV series, Star Wars, and National Geographic provided a deep library to depict both *abundance of content* and *abundance of access*. In contrast, Netflix had the abundance of access but with large production companies removing their content from the streaming service to exclusively stream on their own services, created relative scarcity of content for Netflix. Netflix had foreseen this risk and has been investing upwards of $13 billion since 2018. In other words, while Disney+ can lean on its existing popular IP to create new shows and movies, Netflix does not have that luxury and must budget and experiment with content to stave off the scarcity of both IP and a deep library of existing content.^9^

Finally, the contractual agreements between manufacturers selling products focus on the delivery of material and/or utilization of time. With servitization, the contracts are based on the performance delivered by the service and contractual agreements. The agreements will need to include value cocreation, protection of intellectual property, and service providers are paid on the performance of the product to ensure the effectiveness and efficiency of the outcome for the client (Hypko et al., 2010; Lemley, 2015, 2019; Zhang and Banerji, 2017).

## Conclusion

In this study, we examine that firms (such as Netflix and AWS) delivering services associated with digital products and firms such as Philips associated with physical products are embarking on servitization, that is, they are using their products to sell outcome-as-a-service (Vargo and Lusch, 2008). While as Sklyar et al. (2019) point out “it is possible to servitize without digitizing the offering, and it is possible to digitize the offering without offering it as a service” (p. 456), servitization of digitized products has shifted business models and has created an environment of abundance where there was earlier scarcity. While value was historically derived almost exclusively from scarcity, an environment of abundance increases value while blurring the lines of how that value is measured. Future research may examine how best to create and measure value *via* a servitization model. Overall, this transition has warranted revisiting the operational capabilities of firms failing which the shifting business models may be counterproductive.

## Data availability statement

The original contributions presented in the study are included in the article/supplementary material, further inquiries can be directed to the corresponding author/s.

## Author contributions

The author confirms being the sole contributor of this work and has approved it for publication.

## Conflict of interest

The author declares that the research was conducted in the absence of any commercial or financial relationships that could be construed as a potential conflict of interest.

## Publisher's note

All claims expressed in this article are solely those of the authors and do not necessarily represent those of their affiliated organizations, or those of the publisher, the editors and the reviewers. Any product that may be evaluated in this article, or claim that may be made by its manufacturer, is not guaranteed or endorsed by the publisher.

## Footnotes

^1^Now called Signify.

^2^Available online at: https://markets.businessinsider.com/news/stocks/lighting-as-a-service-disrupts-the-led-lighting-industry-by-enabling-a-market-shift-to-an-opex-model-1027776125.

^3^Available online at: https://ellenmacarthurfoundation.org/topics/circular-economy-introduction/overview.

^4^Available online at: https://www.ledsmagazine.com/leds-ssl-design/modular-light-engines/article/16695809/lighting-as-a-service-poised-to-deliver-the-circular-economy-magazine.

^5^Available online at: https://www.realtynmore.com/signify-launches-interact-iot-platform-in-india/.

^6^Available online at: https://www.sec.gov/Archives/edgar/data/1065280/000106528019000043/form10k_q418.htm.

^7^Available online at: https://www.sec.gov/Archives/edgar/data/1018724/000101872421000004/amzn-20201231.htm.

^8^Available online at: https://www.cnbc.com/2017/08/08/disney-will-pull-its-movies-from-netflix-and-start-its-own-streaming-services.html.

^9^Available online at: https://www.thestreet.com/investing/netflix-has-a-content-problem-not-a-membership-problem.
